# Genomic Instability and Colon Carcinogenesis: From the Perspective of Genes

**DOI:** 10.3389/fonc.2013.00130

**Published:** 2013-05-21

**Authors:** Chinthalapally V. Rao, Hiroshi Y. Yamada

**Affiliations:** ^1^Department of Medicine, University of Oklahoma Health Sciences CenterOklahoma City, OK, USA

**Keywords:** genomic instability, chromosome instability, colon cancer, mice, mitosis, Sgo1, BubR1

## Abstract

Colon cancer is the second most lethal cancer; approximately 600,000 people die of it annually in the world. Colon carcinogenesis generally follows a slow and stepwise process of accumulation of mutations under the influence of environmental and epigenetic factors. To adopt a personalized (tailored) cancer therapy approach and to improve current strategies for prevention, diagnosis, prognosis, and therapy overall, advanced understanding of molecular events associated with colon carcinogenesis is necessary. A contemporary approach that combines genetics, epigenomics, and signaling pathways has revealed many genetic/genomic alterations associated with colon cancer progression and their relationships to a genomic instability phenotype prevalent in colon cancer. In this review, we describe the relationship between gene mutations associated with colon carcinogenesis and a genomic instability phenotype, and we discuss possible clinical applications of genomic instability studies. Colon carcinogenesis is associated with frequent mutations in several pathways that include phosphatidylinositol 3-kinase, adenomatous polyposis coli, p53 (*TP53*), F-box and WD repeat domain containing 7, transforming growth factor*-*β, chromosome cohesion, and *K-RAS*. These genes frequently mutated in pathways affecting colon cancer were designated colon cancer (CAN) genes. Aberrations in major colon CAN genes have a causal relationship to genomic instability. Conversely, genomic instability itself plays a role in colon carcinogenesis in experimental settings, as demonstrated in transgenic mouse models with high genomic instability. Thus, there is a feedback-type relationship between CAN gene mutations and genomic instability. These genetic/genomic studies have led to emerging efforts to apply the knowledge to colon cancer prognosis and to targeted therapy.

## Introduction: Increasing Importance of Molecular Analyses of Colon Cancer

Colorectal cancer (CRC) is a common epithelial neoplasia world-wide, with about 1.2 million newly diagnosed cases and over 600,000 fatalities each year. Tens of millions of individuals identified with colon polyps are at high risk for CRC. In the US, CRC is the second most lethal cancer; 50,830 people are predicted to die of it during 2013 [American Cancer Society (ACS), 2013]. Among all colon cancer cases, approximately 20% have a familial or congenital mutation(s) in gene(s) that increase colon cancer risk, and the cancers tend to develop at an earlier stage of life. The remaining majority (80%) is sporadic, with no obvious genetic causes, and these cases tend to develop later in life, suggesting roles for environmental factors, for time and for accumulation of multiple yet specific genetic mutations and/or for epigenetic alterations. In general, development of sporadic cancer is a slow, age-influenced process with progressive acquisition of genetic mutations and/or epigenetic alterations under the influence of environmental and other external factors. In the colon, normal tissues acquire certain mutations, and develop into hyperplastic epithelia, then into early adenomas. Early adenomas develop into intermediate and late adenomas, then into carcinomas with additional key gene mutations, activation of oncogenes, loss and gain of chromosomes, and/or chromosome amplifications (Fearon and Vogelstein, 1990). This process usually takes decades. Transition from carcinoma-to-metastatic CRC takes an additional 2–3 years.

The cancer stage is well correlated with the current cure rate. Early Stage CRC (stage I and II; localized cancers usually at the adenoma-carcinoma stage) can be cured at a relatively high rate: 80–95% for stage I, 55–80% for stage II. In advanced stages such as metastatic stage IV, the cure rate drops to an unsatisfactory 5–10%. Unfortunately, only around 40% of colorectal cancers are found at the early, relatively curable stages (stages I–II) [American Cancer Society (ACS), 2013]. From the statistics, we can identify several points for improvement and envision approaches to reduce colon cancer-mediated death overall. It is important to note that efficient execution of these approaches requires information regarding the nature of the mutations each colon cancer has acquired. These general approaches and specific examples relevant to CRC include the following: (i) developing methods to identify high-risk groups among the entire population. For CRC, identification of molecular markers that indicate high CRC risk is needed for screening purposes. (ii) Effective use of prevention. In addition to markers, molecular targets for CRC prevention agents (e.g., drugs, natural products, dietary components) need to be identified. (iii) Improvements in screening, detection, and diagnosis for early stage cancers. For CRC detection and screening, visual inspection with colonoscopy is very effective, yet the method is not favored by a majority of patients due to psychological resistance and high cost. Developing a non-invasive marker (e.g., blood marker) would allow development of alternative screening methods. (iv) Addressing unmet clinical needs for therapy. In recent years, it has become possible to monitor molecular markers in a given cancer tissue (e.g., in biopsy samples) and to use the information to make a prognosis or to establish a therapeutic strategy for personalized (tailored) treatments. To perform personalized cancer therapy, information on development and progression of individual cancers is required. For example, in the case of breast cancer, Her-2 status is crucial information for determining applicability of the anti-Her-2 antibody trastuzumab/Herceptin (Tsang and Finn, 2012). Thus there is an increasing need to identify accurate diagnostic and prognostic markers along with therapeutic targets. Contemporary synthetic biological approaches (combining genetics, genomics, proteomics, and bioinformatics) have revealed a great deal of information about the molecular changes in CRC. In this review, we will discuss the relationship between gene mutations associated with colon carcinogenesis and a genomic instability phenotype highly prevalent in colon cancer; and we will discuss emerging translational attempts at prognosis and targeted therapy for colon cancer based on studies of genomic instability.

## The “Vogelgram”

**Figure 1:**
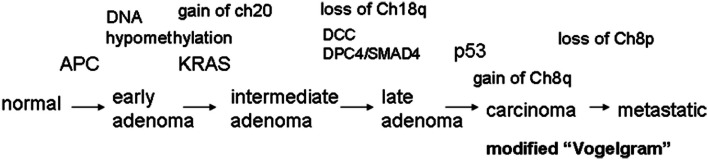
**The “Vogelgram” (modified from the original in Fearon and Vogelstein, 1990)**. The original “Vogelgram” (Fearon and Vogelstein, 1990) mapped loss of chromosome 5q, 12p, 18q, and 17p, and mutations on APC, K-RAS, DCC, and p53 in a sequential order of cancer progression, although the importance of mutation accumulation, rather than sequential order, was emphasized. DNA hypermethylation also was mapped in the early adenoma stage. Later, gain of Chromosome 20 (Davison et al., 2005; Wang et al., 2013), gain of chromosome 8q and loss of Chromosome 8p (Bacolod and Barany, 2010), and mutation in DPC4/SMAD4 (Fleming et al., 2013) were added.

Cancer develops in a stepwise manner, and each step is associated with changes at the molecular level. In 1990, Fearon and Vogelstein proposed a progressive development model of colon cancer and presented some of the key genetic changes associated with the stages of progression (Fearon and Vogelstein, 1990; Grady, 2004) (Figure 1). This type of schematic presentation of the correlation between genetic/genomic changes and stages of colon cancer progression is nicknamed a “Vogelgram.” The model is widely accepted, and newer information from genomics, cytogenetics, and tumor mass sequencing is being added to advance our understanding (e.g., Wood et al., 2007; Chittenden et al., 2008; Brosens et al., 2010). Inactivation of the tumor suppressor Adenomatous polyposis coli (*APC*) is observed at an early stage of colon tumor development. Activation of the *K-RAS* oncogene is associated with transition from early adenoma to intermediate adenoma. Genomic level changes such as loss of chromosome 18, along with loss of Deleted in Colon Cancer (*DCC*) loci, are observed in transition from intermediate adenoma to late adenoma. Loss of tumor suppressor p53 (*TP53*) and gain of chromosome 8q are associated with late adenoma-carcinoma transition. Gaining the ability to metastasize requires additional changes. Loss of chromosome 8p is associated with the carcinoma-to-metastatic transition (Fearon and Vogelstein, 1990; Kinzler and Vogelstein, 1996; Grady, 2004). In the sequence of events, numerical and structural centrosome changes (which would lead to genomic instability; see later section) are observed as early and stable events (Kayser et al., 2005). Since APC mutations that can lead to genomic instability also map as early events, these results suggest that genomic instability begins early in CRC development. Genomic instability has mapped early in ulcerative colitis-related CRC as well (Willenbucher et al., 1999).

## Genomic Instability in Colon Cancer

Cytogenetic studies [e.g., karyotyping, Fluorescence *in situ* hybridization (FISH)] of colon cancers have shown a high degree of genomic instability and aneuploidy. Genomic instability refers to a range of genetic/genomic alterations from point mutations to chromosome rearrangements, whereas aneuploidy is more narrowly defined as having an abnormal number of chromosomes without being polyploid.

There are two major categories of genomic instability in CRC: *Chromosome Instability (CIN)* and *Micro(mini)satellite Instability (MIN)*. CIN is defined mainly from a functional/mechanistic standpoint, and refers to a persistent high rate of chromosome mis-segregation. CIN leads to changes in chromosome number such as chromosome gain or loss. As such, CIN phenotype and aneuploidy refer to a similar or identical condition. In contrast, MIN is often defined from a phenotypical standpoint and refers to repetitive DNA expansions and contractions in the cell. Molecular causes of the MIN phenotype are DNA replication and repair defects. Etiologically, CIN is more prevalent than MIN in CRC. CIN was observed in approximately 85% of colon cancers, and MIN was observed in the remaining 15% (Dunican et al., 2002).

Chromosome instability is caused mainly by failures in the mitotic process (e.g., in chromosome transmission, at the mitotic spindle checkpoint, in kinetochore-microtubule attachment dynamics) and/or in the mitotic apparatus (e.g., kinetochore, centrosome) that cause mitotic process failures. Among a variety of defects that lead to a mitotic failure and CIN, a particularly damaging defect in terms of genomic integrity is centrosome mis-regulation. Centrosome mis-regulation can lead to an abnormal number of centrosomes and multipolar mitosis can occur as a result. As described in later sections, defects leading to centrosome mis-regulation tend to be associated with carcinogenesis [e.g., mutations in p53, Shugoshin 1 (Sgo1)]. In addition, many cell cycle regulators [e.g., breast cancer 1 (BRCA1) and breast cancer 2 (BRCA2; Joukov et al., 2006), Retinoblastoma (Rb; Hernando et al., 2004), forkhead box M1 (FoxM1; Laoukili et al., 2005), RE1-silencing transcription factor (REST; Guardavaccaro et al., 2008); Von Hippel Lindau (VHL; Thoma et al., 2009), Kruppel-like factor (Hagos et al., 2009), Mdm2 (Wang et al., 2007), MdmX (Matijasevic et al., 2008), and RAN binding protein 1 (RanBP1; Tedeschi et al., 2007)] are involved in generation of CIN directly or indirectly. The precise mechanisms are not yet clear in many cases (Thompson et al., 2010).

The MIN phenotype is characterized by repetitive DNA expansions and contractions, and can be caused by defects in DNA replication and repair systems, such as replication slippage, mismatch-repair (MMR) impairment, or homologous recombination defects. MIN is associated with frequent DNA damage or breaks, which can result in gross chromosomal rearrangements such as translocations, duplications, inversions, or deletions. The relationship between MIN and genomic instability was reviewed in more detail elsewhere (e.g., Aguilera and Gómez-González, 2008).

It is to be noted that CIN and MIN are not mutually exclusive. Some cell lines from the NCI 60 panel (60 cell lines derived from cancers of 9 tissue origins that have been invaluable *in vitro* models for cancer research and anti-cancer drug screening) have both CIN and MIN. Examples include KM12, DU-145, SK-MEL-2, and IGROV-1 cell lines (Roschke et al., 2003; Shoemaker, 2006). It is conceivable that these cell lines harbor mutations both in CIN-related gene(s) and in MIN-related gene(s).

Moreover, recent studies on mitotic errors using cell lines revealed that CIN-causing mitotic errors, along with the extended mitotic arrest often associated with them, also can result in DNA damage, chromosomal rearrangements, and chromothripsis – i.e., the fragmentation of a chromosome and its subsequent highly imperfect reassembly (Janssen et al., 2011; Crasta et al., 2012; Orth et al., 2012). A recent report showed that CIN may arise through DNA replication stress (Burrell et al., 2013). Thus, the distinction between CIN and MIN may not be as strict as previously theorized in terms of generation of DNA damage. Further investigation is required to confirm the cell biological observations in animals or humans.

**Figure 2:**
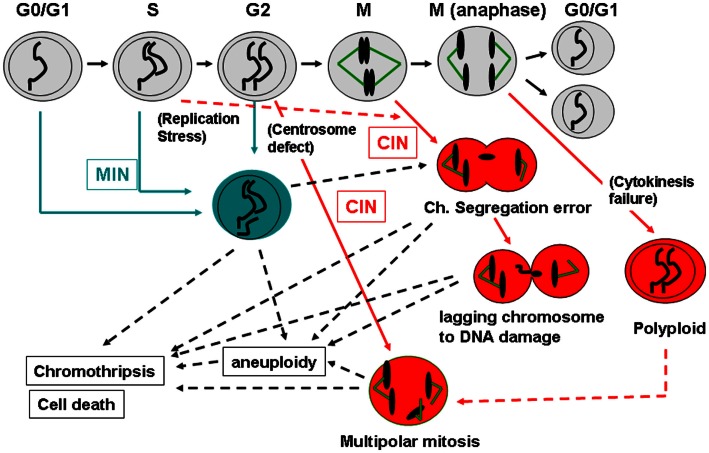
**How MIN and CIN contribute to genomic instability**. Normal cell cycle progression follows G0/G1, S, G2, and M phases (shown in the top with gray-shaded cells). MIN is caused by a defect in DNA repair and/or replication, and is thought to take place mainly during G0/G1, S, or G2 phases in the cell cycle (shown in blue). MIN-type chromosome alteration or its underlying causes also can lead to mitotic errors and CIN (Burrell et al., 2013). CIN is caused by an event in mitosis leading to a chromosome segregation error (e.g., a kinetochore defect, a spindle challenge, a mitotic spindle checkpoint defect, a chromosome cohesion defect; shown in red). CIN also can be caused by an event in a pre-mitotic phase that sets up a mitotic error (e.g., replication stress that can affect mitotic chromosome structure, centrosome mis-regulation that results in multipolar mitosis). CIN-mediated chromosome mis-segregation can lead further to DNA damage, directly caused by cytokinesis machinery or in the process of chromothripsis (Janssen et al., 2011; Crasta et al., 2012). Failures in cytokinesis and subsequent p53-dependent removal of the cell result in generation of a polyploid cell. Polyploidy can lead to aneuploid offspring cells via multipolar mitosis (Shi and King, 2005). Since p53 is involved in centrosome regulation and clustering in addition to removal of aneuploid/polyploid cells, loss of p53 can permit a polyploid-multipolar mitosis-aneuploid cycle, which would be highly detrimental to genomic integrity.

Figure 2 shows a schematic diagram indicating when MIN, CIN, aneuploidy, chromothripsis, and cell death (both cell destruction and senescent/replicative death) occur during the cell cycle. Events causal to the MIN phenotype take place mainly in G1, S, and G2 phases, whereas CIN becomes apparent during mitosis due to an event in mitosis (e.g., a defect in the spindle, the spindle checkpoint, the kinetochore, or chromosomal structure) or in a pre-mitosis phase (e.g., a defect in centrosome integrity or a DNA replication defect in S phase).

In summary, genomic instability in CRC has been subdivided classically into CIN and MIN, with CIN being more prevalent than MIN. Yet, CIN and MIN are not mutually exclusive and can coincide in cancer cells. Newer studies suggest that CIN can lead to not only chromosome loss or gain but to DNA damage and/or to chromothripsis, although it is unclear yet how frequent the events are or how they are tolerated (or eliminated) *in vivo*.

## CAN Genes and Genomic Instability

Tumor mass-sequencing revealed that human colon cancers have several sets of frequently mutated genes and pathways (Wood et al., 2007; Chittenden et al., 2008). The frequently mutated genes were designated as cancer (CAN) genes. The colonic CAN genes/pathways include the signal transducer phosphatidylinositol 3-kinase (*PI3K*), the tumor suppressor/gate keeper *APC*, the tumor suppressor *TP53*, an SCF-E3 ubiquitin ligase complex component F-box/WD repeat-containing protein 7 (*FBXW7*, also known as *hCDC4*), the growth factor Transforming Growth Factor (*TGF*)*-*β, genes involved in chromosome cohesion, and the RAS-GTPase family proto-oncogene *K-RAS* (Wood et al., 2007; Barber et al., 2008; Chittenden et al., 2008). The high frequency of mutations suggests that each pathway plays a significant role in carcinogenesis, and that the mutations function as driver mutations, rather than as secondary passenger mutations. In many cases, the functional significance of the mutations already was demonstrated experimentally in cell biology studies. Each gene/pathway has unique functions in different biological processes. However, surprisingly, in almost all cases (except for *K-RAS*, over which there is controversy), targeted *in vitro* manipulation of these colonic CAN genes [e.g., with small inhibitory (si) or short hairpin (sh)RNA] resulted in elevated genomic instability, directly, or indirectly.

### Phosphatidylinositol 3-kinase

Phosphatidylinositol 3-kinases are a family of related intracellular signal transducing enzymes capable of phosphorylating the three position hydroxyl group of the inositol ring of phosphatidylinositol. The PI3K kinase signaling can be antagonized by the phosphatase/tumor suppressor Phosphatase and tensin homolog (PTEN). Loss of function of PTEN leads to genomic instability through its centrosome interaction (Shen et al., 2007; Liu et al., 2008). Overexpression of oncogenic Met induces centrosome amplification and CIN, and the effect is mediated by PI3K-Akt and p53 (Nam et al., 2010).

### Adenomatous polyposis coli

Mutations in the human *APC* tumor suppressor gene are linked to Familial Adenomatous polyposis (FAP), an inherited cancer-prone condition in which numerous polyps are formed in the epithelium of the large intestine (Kinzler et al., 1991; Kinzler and Vogelstein, 1996; Half et al., 2009). The APC protein functions as a scaffold and physically interacts with a number of proteins relevant to carcinogenesis; thus it acts as a signaling hub. Of particular importance for colonic carcinogenesis is the APC-interacting protein beta-catenin, a Wnt signaling component. When the Wnt pathway is stimulated, beta-catenin activates transcription factor (TCF)-dependent transcription of Wnt-target genes such as Cyclin D1 (*CCND1*), *MYC*, and *EphrinB* (*EphB*), and promotes cell proliferation. Loss of APC influences cell adhesion, cell migration, cytoskeleton, and chromosome segregation (Aoki and Taketo, 2007).

Adenomatous polyposis coli truncations (both MIN^1–850^ and Apc1638T alleles) led to chromosomal instability in mouse embryonic stem cells (Fodde et al., 2001). Binding of APC to microtubules increased microtubule stability *in vivo* and *in vitro*, suggesting a role of APC in microtubule stability (Zumbrunn et al., 2001). APC truncation acted dominantly to interfere with microtubule plus-end attachments and to cause a dramatic increase in mitotic abnormalities (Green and Kaplan, 2003), and the effect was mediated by APC-EB1 interaction (Green et al., 2005; Draviam et al., 2006). Thus, cancer cells with APC mutations have a diminished capacity to correct erroneous kinetochore-microtubule attachments, which would account for the widespread occurrence of CIN in tumors (Bakhoum et al., 2009). In addition, abrogation of the spindle checkpoint function was reported with APC loss of function. Knockdown of APC with siRNA indicated that loss of APC causes loss of mitotic spindle checkpoint function by reducing the association between the kinetochore and checkpoint proteins Bub1 and BubR1, and that it reduces apoptosis and induces polyploidy (Kaplan et al., 2001; Dikovskaya et al., 2007; Rusan and Peifer, 2008). Polyploidy is a major source for aneuploidy since it can lead to multipolar mitosis (Shi and King, 2005). Thus, APC mutation or loss of function can influence CIN in at least three manners: by diminishing kinetochore-microtubule interaction, by the loss of mitotic checkpoint function and by generating polyploid cells.

### p53

Targeted inactivation of p53 in HCT116 cells and in primary human fibroblasts led to no increased rates of numerical or structural chromosomal instabilities, although a tendency toward tetraploidization was observed (a 3.5-fold increase, subtle yet significant) (Bunz et al., 2002). The result suggests that p53 inactivation by itself may have little effect on genomic instability. However, tetraploid cells can be a rich source for aneuploidy, especially if they go through multipolar mitosis subsequently (Shi and King, 2005). p53 Is involved in centrosome clustering and prevents multipolar mitosis in tetraploid cells (Yi et al., 2011). Thus, loss of p53 can increase the risk of genomic instability by generating or permitting tetraploid cells that can go through multipolar mitosis.

In tumors, loss or mutational inactivation of p53 is associated with abnormal amplification of centrosomes (Carroll et al., 1999). Subsequent reports suggest that p53 is involved in the centrosomal duplication cycle, and that the loss of p53 can lead to genomic instability through deregulation of the centrosome duplication cycle and failure to undergo cytokinesis (Tarapore and Fukasawa, 2002; Tomasini et al., 2008). Also, p53 is involved in p21-dependent cell cycle arrest and/or cell death after failed mitosis; thus, the loss of p53 is involved in tolerance of aneuploidy or polyploidy (Tarapore and Fukasawa, 2002; Senovilla et al., 2009; Thompson and Compton, 2010; Vitale et al., 2010).

Therefore, loss of p53 can contribute to genomic instability through at least three pathways: (a) increasing tetraploid cells that can go through multiploar mitosis, (b) centrosomal mis-regulation that also can lead to multipolar mitosis, and (c) permitting survival of aneuploid and tetraploid cells.

### F-box/WD repeat-containing protein 7

F-box/WD repeat-containing protein 7 (also known as FBX7, hCDC4) is the substrate recognition subunit of the SCF-E3 ubiquitin ligase complex, and as such, can target a wide variety of biological processes through ubiquitin/proteasome pathways. The major substrates relevant to carcinogenesis include Cyclin E, MYC, JUN, and Notch (Perez-Losada et al., 2005; Welcker and Clurman, 2008). Genetic inactivation of FBXW7, by means of targeted disruption of the gene in karyotypically stable colorectal cancer cells, results in a phenotype associated with micronuclei and chromosomal instability (Rajagopalan et al., 2004). This phenotype was associated with a defect in the execution of metaphase and subsequent transmission of chromosomes, and was dependent on Cyclin E, a target of FBXW7-SCF-E3 ubiquitin ligase. Deregulation of Cyclin E also is known to cause genomic instability through S-phase delay (Spruck et al., 1999). In breast cancer, high levels of low molecular weight Cyclin E isoforms may contribute to cellular transformation and genomic instability by shortening mitotic progression (Bagheri-Yarmand et al., 2010). Additionally, centrosome duplication during G1/S-phase requires Cyclin E/Cdk2 activity, and Cyclin E mis-regulation contributes to centrosome duplication error (Fukasawa, 2008; Hanashiro et al., 2008; Shimada and Komatsu, 2009). Thus an FBXW7 defect can influence genomic instability through Cyclin E mis-regulation, cell cycle mis-coordination, and centrosome duplication error.

### Transforming growth factor-β

Transforming growth factor-β is a multi-functional cytokine/growth factor. Aberrations of TGF-β signaling have been linked to genomic instability. TGF-β^−/−^ mouse keratinocytes showed aneuploidy and accumulation of chromosomal aberrations, which could be suppressed by the addition of TGF-β in a TGF-β receptor-dependent manner (Glick et al., 1996). v-ras (Ha)-transduced primary TGF-β1^−/−^ keratinocytes and keratinocytes expressing a TGF-β type II dominant-negative receptor transgene have significantly higher frequencies of spontaneous transformation than do control genotypes (Glick et al., 1999). In cervical cancer cells, the introduction of TGF-β1 in the culture medium induced crisis, which was associated with massive chromosomal end-to-end fusions and other structural aberrations, suggesting an involvement of telomere function in TGF-β-mediated CIN (Deng et al., 2008). However, information for the mechanism connecting TGF-beta to CIN or MIN has been largely lacking.

### Chromosome cohesion

Chromosome cohesion is maintained by a ring-shaped cohesin complex that wraps around sister chromosomes, and by other proteins that support the function of or interaction with the cohesin complex, including Sgo1 (“Guardian God” 1 in Japanese) and securin (Oliveira and Nasmyth, 2010). Many yeast CIN mutants were identified later as chromosome cohesion mutants (Thompson et al., 2010). Mutations in cohesin complex subunits in humans are observed in Cornelia de Lange syndrome, a rare, genetically heterogeneous disorder affecting multiple organs and systems during development (Liu and Krantz, 2009). Overexpression of human WAPL protein, a cohesin binding protein, was found in cervical cancers and correlated significantly with the grade of the malignancy (Oikawa et al., 2004, 2008). WAPL involvement in cervical carcinogenesis may be due partially to the resulting chromosomal instability (Ohbayashi et al., 2007). Sgo1 was identified first as a protector of the centromeric cohesin complex from premature degradation or removal during mitosis (Wang and Dai, 2005; Watanabe and Kitajima, 2005). Loss of Sgo1 leads to premature chromosome segregation during mitosis and spindle checkpoint-mediated mitotic delay; it leads to genomic instability in yeast, mice, and human cells (Salic et al., 2004; McGuinness et al., 2005; Wang et al., 2008; Iwaizumi et al., 2009). Later, Sgo1 and cohesin were shown to be involved also in the maintenance of centrosomal integrity (Schöckel et al., 2011).

**Figure 3:**
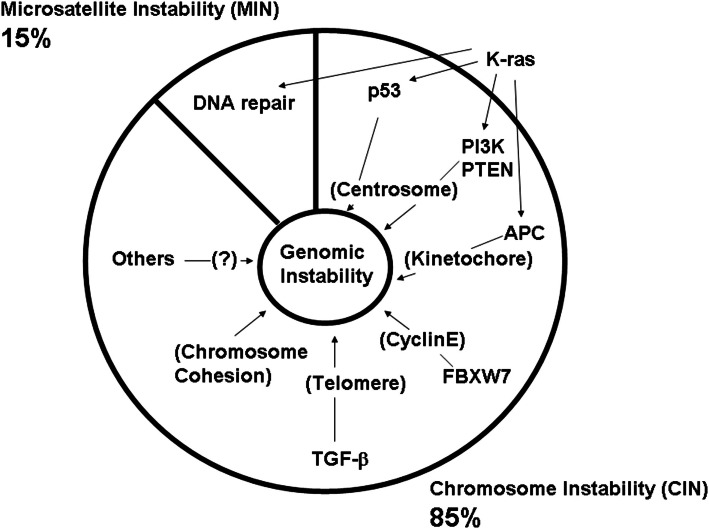
**Defects in major colonic CAN genes are causal to high genomic instability in the colon**. Mass-sequencing projects have identified frequently mutated genes and pathways in colon cancer. They are designated as CAN (cancer) genes (Wood et al., 2007; Chittenden et al., 2008; Brosens et al., 2010). Colonic CAN genes/pathways include p53, PI3K, APC, FBXW7, TGF-β, and chromosome cohesion. Studies have indicated that each CAN gene mutation can lead to genomic instability either by itself or in concert with other mutations (see text). This effect would explain why nearly all advanced colon cancers show a high degree of genomic instability.

Thus, normal functions of these frequently mutated CAN genes are directly or indirectly involved in genomic fitness and prevention of CIN. Abnormalities in chromosome structure, and in structure and function of the mitotic apparatus (e.g., centrosome, kinetochore, telomere), often are caused by aberrant CAN gene functions (Figure 3; Table 1).

**Table 1:** **Cancer genes and pathways involved in CIN**.

Major colonic CAN genes/pathways	Function	Involvement in CIN
p53	Transcription factor	Tetraploid generation (Bunz et al., 2002)
	Tumor suppressor	Centrosome mis-regulation (Carroll et al., 1999; Tarapore and Fukasawa, 2002; Tomasini et al., 2008)
		Tolerance to aneuploidy/polyploidy (Tarapore and Fukasawa, 2002; Senovilla et al., 2009; Thompson and Compton, 2010; Vitale et al., 2010; Yi et al., 2011)
PI3K/PTEN	Signal transduction kinase/phosphatase	Centrosome mis-regulation (Shen et al., 2007; Liu et al., 2008; Nam et al., 2010)
APC	Scaffold protein, signaling hub	Kinetochore-microtubule attachment (Green and Kaplan, 2003; Green et al., 2005; Draviam et al., 2006; Bakhoum et al., 2009)
		Spindle checkpoint defect (Kaplan et al., 2001; Dikovskaya et al., 2007; Rusan and Peifer, 2008)
		Polyploid cell generation (Dikovskaya et al., 2007; Rusan and Peifer, 2008)
FBXW7	A component of SCF ubiquitin ligase complex	Centrosome mis-regulation via Cyclin E (Fukasawa, 2008; Hanashiro et al., 2008; Shimada and Komatsu, 2009)
		Transcription factor mis-regulation (Perez-Losada et al., 2005; Welcker and Clurman, 2008)
		Cell cycle mis-regulation (Spruck et al., 1999; Bagheri-Yarmand et al., 2010)
TGF-β	Cytokine, Growth factor	Telomere dysfunction (Deng et al., 2008)
Chromosome cohesion (cohesins, Sgo1)	Chromosome structure, centriole engagement/centrosome integrity	Premature Chromosome separation (Salic et al., 2004; McGuinness et al., 2005; Wang et al., 2008; Iwaizumi et al., 2009; Yamada et al., 2012)
		Centrosome mis-regulation (Schöckel et al., 2011; Yamada et al., 2012)
K-RAS	Signaling GTPase, protein modification	Accelerate carcinogenesis in concert with other mutations (Hingorani et al., 2005; Luo et al., 2007, 2009, 2011)
		May cause genomic instability by itself (H-RAS studies: Ichikawa et al., 1990; Denko et al., 1994; Saavedra et al., 2000; Woo and Poon, 2004; Knauf et al., 2006)

### K-RAS

In contrast to other prominent CAN genes, a direct connection of K-RAS activation to genomic instability has been controversial. Several experiments using cell lines to express activated H-RAS showed an increase in genomic instability (Ichikawa et al., 1990; Denko et al., 1994; Saavedra et al., 2000; Woo and Poon, 2004; Knauf et al., 2006), suggesting that expression of activated K-RAS (which presumably functions in a very similar or identical manner to the isoform H-RAS) (Baker et al., 2013) may have a similar effect and be able to promote genomic instability by itself. However, the role of K-RAS *in vivo* in carcinogenesis and in genomic instability has remained unclear, in part because the experiment is not as straightforward as knockdown or inhibition experiments (Castagnola and Giaretti, 2005). Some transgenic mouse-based studies tackled this question. Luo et al. (2011) reported on the combined effect of treatment with the DNA-alkylating colon carcinogen DMH and *Cre*-*LoxP-*mediated K-rasG12D activation in colon carcinogenesis in mice, and concluded that mutant K-ras significantly promotes DMH-induced colorectal carcinogenesis, resulting in distinct changes in cell signaling and proliferation, but that it does not alter chromosome stability in the tumors. Yet, another study showed that combining mutations in p53 (Trp53^R172H^) and K-RAS (G12D) in a PDX-1-Cre pancreatic cancer mouse model resulted in rapid development of invasive and metastatic pancreatic ductal adenocarcinoma with a high degree of genomic instability. The genomic instability was accompanied by frequent centrosomal defects and multipolar mitosis, which may have been caused by the p53 defect (Hingorani et al., 2005). Thus, K-RAS activation at least exacerbated genomic instability in the pancreas. Other mouse model studies have shown that mutant K-*ras* accelerates intestinal carcinogenesis on both a mutant *Apc* background and an *Mut S Homolog 2* (*Msh2*)-null background (Luo et al., 2007, 2009). Since both *APC* and *Msh2* mutations can cause genomic instability by themselves (*Apc*: CIN; *Msh2*: MIN), a role of K-ras mutation in the gastrointestinal (GI) tract may be at least to exacerbate already existing genomic instability, although K-*ras* mutation alone may be insufficient to generate tumor aneuploidy. The differing results between tumors of the GI tract and pancreas may be a matter of organ specificity.

In summary, most of the frequently mutated genes and pathways involved in colon carcinogenesis (colonic CAN genes) are involved in genomic instability or, in the case of K-ras, at least exacerbate it.

## Insights from Transgenic Mouse Models

*In vitro* studies described above support the notion that aberrations in major colonic CAN genes lead to or facilitate genomic instability. Is genomic instability itself causal to and/or enhancing of colon carcinogenesis *in vivo*? To address this question, several transgenic mouse models were generated in which genes involved in the processes associated with genomic instability were targeted to test for effects on carcinogenesis (Foijer et al., 2008; Ricke et al., 2008; Rao et al., 2009; Schvartzman et al., 2010).

## Apc Mouse Model and Colon Cancer

The first mouse model that contained a mutation in the Apc gene was designated multiple intestinal neoplasia (Min) (Su et al., 1992). This mouse was obtained in *N*-ethyl-*N*-nitrosourea an (ENU) mutagenesis screen. Min mice were found to have a nonsense mutation at the region corresponding to codon 850 of the Apc gene. The Min mutation results in a truncated protein of 850 amino acids. Apc Min heterozygotes (Apc^min/+^) are born normally but have a reduced average lifespan of 150 days. These mice can develop more than 100 adenomas in the small intestine, depending on the genetic background. On average, the mice developed between 18 and 62 tumors per mouse in the small intestine but only 0–4 tumors per mouse in the colon. All of the histopathologically classified tumors in the small intestine and those in the colon were adenomas (adenomatous polyps), with no evidence of local invasion of the lamina propria. The Apc^min/+^ mouse model has been used in numerous studies of GI tract carcinogenesis, alone or in combination with other mutations or drugs (Taketo, 2006; Rosenberg et al., 2009; Taketo and Edelmann, 2009).

## Micro(mini)satellite Instability Models and Colon Cancer

Micro(mini)satellite instability is observed in a significant proportion (15%) of human colon cancer. Up to 5% of human colon cancers diagnosed in the US are categorized as hereditary non-polyposis colorectal cancer (HNPCC) with MIN phenotype, and causal gene mutations have been identified. Mutations in the two human DNA repair genes *MSH2* and *Mut L Homolog 1* (*MLH1*) are responsible for HNPCC, as well as for a significant number of sporadic colorectal cancers with MIN (Kinzler and Vogelstein, 1996; Half et al., 2009). The observation raises questions about the role of these DNA repair proteins and the homologs in the initiation and progression of colorectal cancer. To address these questions, mice with inactivating mutations in all of the known *mutS* and *mutL* homologs have been generated (i.e., Msh2, Msh3, Msh4, Msh5, Msh6, Mlh1, Pms1, and Pms2; Heyer et al., 1999). Analyses of the mouse phenotypes revealed a role for some of the DNA mismatch-repair genes in lymphoma and GI tract carcinogenesis and in mammalian meiosis. Among the transgenic mutant mice, msh2, msh3, msh6, and mlh1 mice showed cancer development in the GI tract.

### Mut S homolog 2

Mut S homolog 2-deficient mice are fertile and develop normally. Msh2 homozygous mutant mice (^−/−^) have a reduced life span compared with wild type and heterozygote (^−/+^) mutant animals. Fifty percent of the animals die by the age of 6 months and all animals were dead by 12 months of age due to T-cell lymphomas. Those MSH2-deficient mice that survive more than 6 months develop GI and skin tumors. The GI tumors found in these studies were classified as adenomas and carcinomas (Reitmair et al., 1996).

### Msh6 and Msh3

In human cells, the two protein complexes consisting of MSH2–MSH3 and MSH2–MSH6 appear to be responsible for the recognition of mispaired bases during MMR. The MSH2–MSH6 complex recognizes single nucleotide and small insertion/deletion mismatches, and the MSH2–MSH3 complex recognizes small insertion/deletion mismatches (Guerrette et al., 1998). Mice deficient in MSH6 are fertile and develop a cancer susceptibility syndrome similar to Msh2 mutant mice (Edelmann et al., 1997). A majority of these mice develop invasive B and T-cell lymphomas and GI tumors within their first year. A surprising difference between Msh2- and Msh6-deficient mice is the lack of microsatellite instability in Msh6-deficient tumors. The MIN analysis data implies that the Msh6 defect-mediated MIN by itself may not be responsible for tumorigenesis but that it may provide cancer predisposition (Edelmann et al., 1997).

Cells from Msh3^−/−^ mice are defective in repair of insertion/deletion mismatches but can repair base-base mismatches. The survival rate of Msh3^−/−^ mice was not significantly different from that of wild type, though the Msh3^−/−^ mice developed tumors at a late age (Edelmann et al., 2000). Msh3^−/−^ msh6 double mutant mice showed a GI tract cancer-prone phenotype nearly identical to that of msh2 mice, indicating functional overlap between msh3 and msh6 proteins (de Wind et al., 1999; Edelmann et al., 2000).

### Mut L homolog 1

Targeted inactivation of the mutL homolog Mlh1 in mice leads to infertility and tumor susceptibility. Fifty percent of the MLH1-deficient animals die prior to 6 months of age and all animals die by 13 months of age because of the development of T-cell lymphomas and GI tumors. GI tumors were found throughout the small intestine and ranged from adenomas to early invasive carcinomas. Typically, one to two GI tumors were found per mouse (Edelmann et al., 1999). Cases of a human MLH1 kindred that carried a homozygous MLH1 mutation also were reported. These patients died at a very young age and developed leukemias and/or lymphomas and neurofibromatosis type I, showing resemblance to the mouse phenotype (Ricciardone et al., 1999; Wang et al., 1999).

Hegan et al., compared the degree of genomic instability in mice deficient for *Pms2*, *Mlh1*, *Msh2*, *Msh3*, or *Msh6* or both *Msh2* and *Msh3* or both *Msh3* and *Msh6* using two reporter gene systems. Among the single nullizygous (^−/−^) mice, Mlh1, and Msh2 deficiency produced the greatest instability, whereas Msh3 deficiency generated the least. Compared with wild type, the double mutant mice deficient for both Msh2 and Msh3 or deficient for both Msh3 and Msh6 displayed the largest increases in mutation frequencies of all the groups (Hegan et al., 2006). Thus, at least in some cases, the degree of genomic instability is correlated with GI tract cancer predisposition *in vivo*.

## Chromosome Instability Models and Colon Cancer

Chromosome instability model mice were generated by targeting mitotic processes. The targets included mitotic spindle checkpoint components Mad1 (Iwanaga et al., 2007), Mad2 (Dobles et al., 2000; Michel et al., 2001), Bub1 (Baker et al., 2009; Baker and van Deursen, 2010; Ricke et al., 2011, 2012), BubR1 (Baker et al., 2004; Wang et al., 2004), Bub3 (Kalitsis et al., 2000), mitotic motor CenpE (Weaver et al., 2007), and chromosome cohesion/centrosome integrity protector Sgo1 (Wang et al., 2008; Yamada et al., 2012) (reviewed in Foijer et al., 2008; Ricke et al., 2008; Rao et al., 2009; Schvartzman et al., 2010). As predicted, the transgenic mice commonly showed an increase in genomic instability at the cellular level, demonstrated via fibroblast culture, blood cell karyotyping, and/or FISH analyses of tissues. Cancer-prone phenotypes were observed, especially in liver and lung, albeit the degree was modest in most strains. Although each targeted gene may have an additional role outside of mitosis, the results collectively point to a conclusion that CIN and aneuploidy can be causal to carcinogenesis. A CenpE mutation functioned as both an oncogenic and a tumor suppressor, suggesting that carcinogenesis in the models is a result of imbalance between pro-carcinogenic and anti-carcinogenic effects rather than from a straightforward drive toward cancer (Weaver et al., 2007). The duality may provide a proof-of-principle for cancer therapy that targets the mitotic checkpoint and elevates chromosome mis-segregation (Janssen et al., 2009; Colombo and Moll, 2010). The relationship to carcinogenesis in the GI tract was investigated in two CIN strains, BubR1 and Sgo1.

### BubR1

Human BubR1 is an essential component of the mitotic spindle checkpoint (Chan et al., 1999). Congenital mutation in the *BubR1* gene was linked to mosaic variegated aneuploidy (MVA), a rare cancer-susceptible disorder (Hanks et al., 2004; Suijkerbuijk et al., 2010). In mice, BubR1 complete knockout results in death *in utero* before E8.5 (Dai et al., 2004). Depending on the allele and the degree of the loss of the function, the mouse phenotype varies. Mice with a viable yet strong loss of function allele BubR1^H/H^, which expresses only 10–20% of BubR1 protein compared with wild type, demonstrated that near complete loss of BubR1 function results in cellular senescence and a premature aging phenotype at the whole animal level (Baker et al., 2004; Matsumoto et al., 2007). The premature aging is at least in part dependent on p16^INK4A^ expression (Baker et al., 2008); some of the premature aging phenotypes could be reversed by selectively eliminating p16-expressing cells from tissues (Baker et al., 2011). Mice with weaker alleles such as the haploinsufficient (BubR1^−/+^), which expresses about 50% of the protein, developed apparently normally and showed only a modest phenotype, although reduction of overall lifespan was reported (Dai et al., 2004; Wijshake et al., 2012). When challenged with Azoxymethane (AOM), a commonly used carcinogen for colon cancer studies, BubR1^−/+^ mice showed significantly higher number and larger size of Aberrant Crypt Foci (ACF), colonic precancerous lesions, than did wild-type control; and they developed colon tumors (microadenoma) (Dai et al., 2004). When combined with APC^min/+^, BubR1^−/+^ mice showed a higher number of tumors in the colon (Rao et al., 2005). These studies indicated that the haploinsufficiency significantly enhances carcinogenesis, although BubR1 haploinsufficiency by itself may not be a strong driver of carcinogenesis in the GI tract.

### Shugoshin 1

Studies in yeast and humans demonstrated that a function of Sgo1 is as a protector of centromeric cohesion of mitotic chromosomes, and that the loss of function results in genomic instability through premature chromosome separation (Salic et al., 2004; McGuinness et al., 2005; Wang et al., 2008; Iwaizumi et al., 2009). Later, an unexpected role of Cohesin-Sgo1 complexes in centrosomal integrity was demonstrated. A Cohesin-Sgo1 complex localized to the centriole (a core structure of the centrosome) was involved in engagement of the centriole, and Sgo1 depletion led to centriole disengagement (Schöckel et al., 2011). In human colon cancers, Sgo1 mRNA level is decreased, and siRNA-mediated inhibition of Sgo1 in the colon cancer cell line HCT116 resulted in genomic instability (Iwaizumi et al., 2009). Chromosome cohesion, a main process that Sgo1 influences, is frequently defective in colon cancer (Barber et al., 2008). Sgo1^−/+^ mice developed apparently normally (Wang et al., 2008). However, Sgo1 Mouse Embryonic Fibroblasts (MEF) showed phenotypes reflecting the dual functions of Sgo1, namely mitotic errors consistent with loss of chromosome cohesion, and multiple centrosomes, consistent with a function in centrosome integrity (Yamada et al., 2012). When challenged with the carcinogen AOM, as in BubR1^−/+^ mice, Sgo1^−/+^ mice showed significantly higher number and larger size of colonic precancerous ACF lesions than did wild-type control, and they developed more tumors (Yamada et al., 2012). Interaction between APC and Sgo1 mutations is being investigated (Yamada et al., unpublished).

Results from these two CIN mouse models indicate that the mutations that create mitotic error-induced genomic instability can enhance carcinogenesis in the GI tract.

## Why is CIN So Prevalent in CRC?

The high prevalence of CIN in colon cancer suggests two non-mutually exclusive possibilities: (i) gene mutations causal to colonic carcinogenesis are involved in CIN, and (ii) CIN by itself may be either causal or promotional to colon carcinogenesis. As discussed in previous sections, both possibilities have supporting evidence. For the second possibility, some mechanistic insights have been offered from mouse models. Once a high CIN condition is introduced by mutation in the mitotic spindle checkpoint gene Bub1 in mice, loss of chromosomes, and/or loss of heterozygosity (LOH) of tumor suppressors is accelerated (Baker et al., 2009). Such events would contribute to and/or facilitate carcinogenesis directly.

In addition, by applying a concept from microbiology, it has been argued that a high CIN condition may provide an adaptive advantage in cancer evolution (Chandhok and Pellman, 2009). With high CIN, a higher level of mutation and diversity is introduced to cellular offspring, which would serve to adapt for survival, as long as the new mutations do not immediately kill the cells. Consistent with this notion, Benezra’s group first induced lung tumors in mice via lung-specific doxycycline-mediated oncogenic K-RAS activation; then, by shutting down the K-RAS expression, they created a condition that mimics oncogene withdrawal and resulted in tumor shrinkage and remission. The tumors recur with a modest rate in a wild-type background. However, in mice haploinsufficient for the spindle checkpoint component Mad2 that show high genomic instability, a higher lung tumor recurrence rate was observed (Sotillo et al., 2010). Several studies of human cancers also concluded that patients with colon cancer with high CIN show significantly poorer survival compared with patients with colon cancer with low CIN or with high MIN (Watanabe et al., 2012). Thus, high CIN is associated with or even causal to high cancer recurrence and poor survival.

## Practical Applications of Genomic Instability Studies

In general, how humans and animals handle genomic instability is still quite poorly characterized. Cells with a very high degree of genomic instability may not be compatible with life; they may go through destruction (e.g., apoptotic or necrotic death, phagocytosis) or replicative death (e.g., senescence). However, cells with modest genomic instability can survive. Such cells, particularly those with progenitor/stem cell lineage, may be a latent source of cancer if they are given the opportunity to accumulate further mutations. Can we detect the presence of such cells? Can such cells be removed selectively? The following section of this review describes emerging approaches for addressing these questions:

(i) **Gene markers indicative of genomic instability for screening and prognosis**
  High genomic instability is associated with gene expression changes. Conversely, certain gene expression changes can predict the presence of a high degree of genomic instability and can serve as genomic instability markers, which can be detected with PCR-based tests. Carter et al., identified a signature of chromosomal instability from specific genes whose expression was consistently correlated with total functional aneuploidy in several human cancer types. Net overexpression of this signature was predictive of poor clinical outcome in 12 cancer data sets (Carter et al., 2006). Similarly, with microarray-based expression analysis, Habermann et al. (2009) identified 12-gene expression signatures for genomic instability in breast cancer. The authors used gene expression profiling of 48 breast cancer specimens that differed profoundly in their degree of genomic instability and identified a set of 12-genes that define the two groups (genome stability vs. genomic instability). The gene signatures include: nuclear RNA export factor 1 (NXF1), cDNA DKFZp762M127, p28 (dynein, axonemal, light intermediate chain 1), KIAA0882, v-myb, CDKN2A (p16^INK4A^), RAS-like, estrogen-regulated, growth inhibitor (RERG), chemokine (C–C motif) ligand 18 (SCYA18), aurora kinase A (STK15), forkhead box A1 (HNF3A), and two unknown genes (Habermann et al., 2009; Mettu et al., 2010). Expression of the gene signatures was scored with a tumor database, and a significant correlation between high expression of the signatures and poor prognosis in breast cancer was shown. In subsequent analysis, the 12-gene signature was tested with ovarian, small cell lung carcinoma, and colorectal cancers. In all three types of cancers, there was significant correlation between the signature expression and cancer recurrence, suggested by Kaplan–Meier survival curves over a 15-year period (Mettu et al., 2010). Thus, the gene signatures may have application as universal genomic instability markers. The functional significance of the marker expression needs to be investigated, and validation of the markers as drug targets needs to be conducted for further translational application. However, this information for CIN-associated gene expression signatures can be used to develop a PCR-based gene expression analysis kit applied to biopsy tissues (cancerous biopsy specimens for prognosis, and possibly normal-looking tissues for risk prediction).
(ii) **Emerging attempts to target aneuploidy for cancer therapy**
  Aneuploidy is a form of genomic instability and a result of CIN. Since genomic instability and aneuploidy are prevalent among cancers, a notion has developed that targeting cells with aneuploidy or genomic instability will selectively eliminate tumor initiating cancer cells. Amon’s group has been characterizing aneuploid cells in yeast and in mice, and found that aneuploidy leads to cell proliferation defects as well as proteotoxic and energy stress (Williams et al., 2008; Tang et al., 2011; Oromendia et al., 2012). The authors rationalized that additional drug-mediated interference with pathways that already are impaired in aneuploidy or that are essential for cell viability may lead to lethality (synthetic lethal approach). They demonstrated that 17-AAG, a drug that inhibits heat shock protein 90 (HSP90), and aminoimidazole carboxamide ribonucleotide (AICAR), a drug that induces adenosine monophosphate kinase (AMPK) activation, show aneuploid cell-specific efficacy through p53-dependent cell death (Tang et al., 2011). With independent lead studies, several HSP90 inhibitors including 17-AAG have entered Phase I/II clinical trials and the evaluation is ongoing (Jhaveri and Modi, 2012; Neckers and Workman, 2012). These drugs may prove effective against cancers that generate a high degree of aneuploidy without harming normal cells by exploiting specific weaknesses in aneuploid cells.

## Conclusion

It has been over a century since Theodore Boveri proposed a relationship between carcinogenesis and aneuploidy in the early twentieth century (Boveri, 2008; Holland and Cleveland, 2009). During the past century, a number of genes involved in colonic carcinogenesis and progression have been identified, and their roles in generating genomic instability have been demonstrated, at least in part. Transgenic mouse models have indicated that mutations that create genomic instability also can be carcinogenic; and novel use of the knowledge from genomic instability studies has begun to be explored. Although colon cancer is a major lethal cancer at this moment, this situation should change in the future with continuing efforts to translate basic science to the bedside.

## Conflict of Interest Statement

The authors declare that the research was conducted in the absence of any commercial or financial relationships that could be construed as a potential conflict of interest.
